# Impacts of risk assessment data, assumptions, and methods: Considering the evidence for diacetyl and 2,3-pentanedione

**DOI:** 10.3389/fpubh.2022.972136

**Published:** 2022-09-08

**Authors:** Douglas O. Johns, Christine Whittaker, Jean M. Cox-Ganser

**Affiliations:** ^1^Spokane Mining Research Division, Centers for Disease Control and Prevention, National Institute for Occupational Safety and Health, Spokane, WA, United States; ^2^Division of Science Integration, Centers for Disease Control and Prevention, National Institute for Occupational Safety and Health, Cincinnati, OH, United States; ^3^Respiratory Health Division, Centers for Disease Control and Prevention, National Institute for Occupational Safety and Health, Morgantown, WV, United States

**Keywords:** risk assessment, occupational exposures, respiratory health, α-diketones, coffee roasting and packaging

## Abstract

The articles published as part of the *Frontiers in Public Health* research topic, “Investigating exposures and respiratory health in coffee workers” present research findings that better characterize exposures to diacetyl and 2,3-pentanedione and inform our understanding of the health risks posed by these exposures. Although various research groups and organizations have conducted risk assessments to derive occupational exposure limits (OELs) for diacetyl, differences in the data used and assumptions made in these efforts have resulted in a wide range of recommended OELs designed to protect human health. The primary drivers of these differences include the decision to use data from human or animal studies in conducting a quantitative risk assessment, and the application of uncertainty factors (UF) to derive an OEL. This Perspectives paper will discuss the practical implications of these decisions, and present additional commentary on the potential role that the recent investigation of human exposures to relatively low concentrations of α-diketones, specifically diacetyl and 2,3-pentanedione, may play in supporting qualitative or quantitative human health risk assessments.

## Introduction

In 2016, the U.S. National Institute for Occupational Safety and Health (NIOSH) recommended an 8-hour time-weighted average (TWA) occupational exposure limit (OEL) for diacetyl (2,3-butanedione) of 5 ppb. OELs can take various forms, but generally speaking are all science- and health-based upper limits of exposure derived by government and professional organizations to protect worker health. NIOSH's recommended exposure limit (REL) for diacetyl was supported and informed by an extensive and comprehensive review and analysis of the scientific literature, including qualitative and quantitative risk assessments from animal toxicological and human epidemiologic investigations (1). NIOSH also established a REL for 2,3-pentanedione (acetylpropionyl) of 9.3 ppb, similar but slightly higher to that of diacetyl, owing to their structural similarities while also considering limitations of the analytical method for 2,3-pentanedione. The risk assessments for diacetyl and 2,3-pentanedione were subject to peer and stakeholder review and public comment. Other researchers and organizations have conducted and published risk assessments for diacetyl, prior and subsequent to the publication of the NIOSH criteria document. The authors of these assessments have recommended OELs for diacetyl ranging from 1 to 200 ppb (0.001 to 0.2 ppm) (2–5).

What accounts for this variability between OELs? In truth, such a wide range – greater than two orders of magnitude – is not atypical, and is a function of a number of factors that differ among assessments including: (1) selection of different health endpoints upon which to base an assessment, (2) use of different types of data, such as that from animal laboratory studies vs. data from human observational investigations to derive OELs, (3) methods for estimating exposures in epidemiologic investigations, (4) applying different protocols for interspecies extrapolations – for example, using allometric scaling, incorporating pharmacokinetic information, or using different inhalation dosimetry methods, (5) new data and changes over time in perceived acceptable levels of risk, and (6) efforts to adequately address uncertainty, variability and confounding. Ideally, differences in methods are adequately described in the peer-reviewed scientific literature and will lead to healthy dialogue and scientific debate. Increasing transparency helps to reduce, but does not completely eliminate, confirmation biases of individual scientists – a common tendency to seek answers that support preexisting views or hypotheses (6).

When new high quality data become available or new analytical methods are developed, they may cast doubt or improve the confidence of prior risk assessments. In these cases, risk assessors may conduct additional analyses or new assessments to improve our understanding of health effects resulting from occupational exposures. A recently published series of articles describes investigations of occupational exposures, focusing on α-diketones, and respiratory health among workers employed at small and medium-sized coffee roasting and packaging facilities (7). Diacetyl and 2,3-pentanedione exposures in these investigations were found to exceed the NIOSH REL, particularly among groups of workers whose tasks included grinding, flavoring, and packaging coffee (8). However, these exposures were typically far lower (one to greater than two orders of magnitude) than the exposures observed among microwave popcorn workers upon which NIOSH based its quantitative risk assessment for diacetyl (1).

This perspectives paper will briefly describe published quantitative risk assessments for diacetyl and consider how the recently reported findings of exposures among coffee workers may impact the understanding of occupational risks from exposure to diacetyl as well as 2,3-pentanedione. Note, a list of acronyms used throughout this paper is presented in Table 1 along with their meaning.

**Table 1:** List of acronyms.

**Acronym**	**Full form**
EPA	United States Environmental Protection Agency
FEV_1_	Forced expiratory volume in 1 s
FEV_1_/FVC	Ratio of FEV_1_ to forced vital capacity
LOAEC	Lowest observed adverse effect concentration
NIOSH	The National Institute for Occupational Safety and Health
NTP	National Toxicology Program
OEL	Occupational exposure limit
ppb	Parts per billion
ppm	Parts per million
ppm-yr	Parts per million - years
REL	NIOSH recommended exposure limit
SCOEL	European Commission's Scientific Committee on Occupational Exposure Limits
TWA	Time weighted average
UF	Uncertainty factor

### Quantitative risk assessments for diacetyl

Several investigators have conducted quantitative risk assessments for occupational exposure to diacetyl, the details of which are briefly summarized in Table 2. The differences in the recommended OELs derived from these risk assessments are largely due to differences in data used and application of uncertainty factors (UF) or extrapolation to an occupational lifetime exposure (e.g., 8 h per day, 5 days per week for 45 years).

**Table 2:** Risk assessments based on human and animal studies of respiratory health effects from diacetyl exposure.

**Risk assessment citation**	**Health endpoint**	**Risk assessment methods and assumptions**	**Derived OEL**
**Human based assessments**			
SCOEL (5)	Changes in pulmonary function, symptoms, and other respiratory health endpoints in exposed workers.	Using Haber's Law, calculated the 40-year working lifetime concentration corresponding to the lowest observed adverse effect concentration (LOAEC). Corrected for bias in exposure estimation and applied an UF of 2.	0.02 ppm
NIOSH (1)	Changes in pulmonary function, symptoms, and other respiratory health endpoints in exposed workers.	Assumed cumulative exposures over a 45-year working lifetime using regression modeling. Targeted excess risk level of 1 case per 1,000 workers exposed over a working lifetime. No additional UF applied.	5 ppb
Egilman et al. (3)	Changes in pulmonary function in exposed workers (cross-sectional design).	Assumed cumulative exposures over working lifetime of 45 years. Divided exposure estimates by the attributable increased risk to achieve a rate ratio of 1.0 and then extrapolated exposure estimates to working lifetime to estimate safe levels. No additional UF applied.	1 ppb
**Risk assessment citation**	**Health endpoint**	**Risk assessment methods and assumptions**	**Derived OEL** *
**Animal based risk assessments**			
Maier et al. (4)	Tracheobronchial inflammation in mice after subchronic exposure.	Combined data from 6- and 12-week studies; Estimated excess risk using benchmark dose techniques, extrapolated to human equivalent concentration using EPA regional gas dose ratio method refined with computational fluid dynamics model developed for rats and humans. Applied an UF of 10.	0.2 ppm
NIOSH (1)	Lung inflammation in male rats after subchronic exposure.	Estimated excess risk using benchmark dose techniques, extrapolated to human equivalent concentration using EPA regional gas dose ratio method and pharmacokinetic modeling. Applied an UF of 24.	0.06 ppm*
Beckett et al. (2)	Bronchiolar hyperplasia in rats after chronic exposure.	Estimated excess risk using benchmark dose techniques, extrapolated to human equivalent concentration using EPA regional gas dose ratio method and pharmacokinetic modeling. Applied an UF of 8.	0.2 ppm

* The final NIOSH REL was based on human data and the REL derived from animal data is included for comparison (1).

Of note, the human-based risk estimates were all lower than the lowest animal-based risk estimates. In the worker exposure-based risk assessments, the European Commission's Scientific Committee on Occupational Exposure Limits (SCOEL) (5) used straightforward extrapolation to a working lifetime (40-year) exposure from Kreiss et al. (9) adjusted for an UF of 2 (after a correction for bias in the exposure assessment). The NIOSH risk assessment used data from Kreiss et al. (9); Kullman et al. (10); Kanwal et al. (11); and Kanwal et al. (12) with risks estimated using regression techniques to extrapolate to a working lifetime (45-year) exposure. Finally, Egilman et al. (3) used data from Lockey et al. (13), extrapolated to a 45-year working lifetime and applied a safety factor equal to the excess risk rate in the exposed group. The observed differences in risk among the epidemiologic-based risk assessments, then, were driven largely by selection of critical data set and method of extrapolation or selection of UF, which underscores the critical importance of these decisions in characterizing occupational risks.

The three animal-based assessments used three different studies as their primary data source. Maier et al. (4) was based on a 6- and 12-week study with fewer animals than the other two, while NIOSH (1) was based on a subchronic (90-day) study and Beckett et al. (2) was based on a 2-year bioassay. In comparing the proposed OELs from Beckett and NIOSH, the approximate factor of 3 difference between them appears to be almost entirely due to a selection of UF of 8 by Beckett and 24 by NIOSH, corresponding to the difference in primary study length. Beckett selected an UF which comprised an animal to human factor of 2.5 for toxicodynamic differences (also called variability in susceptibility) and an interindividual (human) factor of 3.2 for toxicodynamic differences between individuals, which, when multiplied, equals a composite UF of 8. NIOSH, working from the subchronic study, rather than the 2-year bioassay, applied the identical factors with an additional UF of 3 for the extrapolation from a subchronic study to a lifetime study. The Maier et al. (4) study was based on a smaller study combining a 6-week and 12-week dataset, using an UF of 10.

Several factors may have contributed to the observed differences in the risk estimates for diacetyl, including selection of animal-based or worker-based data sets, and there are advantages and disadvantages to using animal data or human data. In animal studies, there is certainty about the exposures (although typically higher than human exposures), a homogenous population, and few confounding factors to consider. With human data, exposures are estimated in the species of concern to risk assessors (humans), but exposures are often estimated with wide confidence limits and may be confounded by other exposures and other factors that can influence exposures or health effects of concern. In animal-based risk assessments, uncertainty factors are typically applied to account for interspecies and inter-individual differences in susceptibility and when data on metabolism and disposition of a chemical are not available. When human data are available, risk assessors may apply uncertainty factors to measured exposures or conduct regression analyses to extrapolate risks to working lifetime exposures. In the case of diacetyl, NIOSH used the epidemiological data to estimate the risk of changes in lung function beyond the range of the available data to a working lifetime, assuming that chronic exposure to low levels of diacetyl would result in accumulated and persistent damage. In the absence of evidence to the contrary, this health protective assumption impacts how risks are determined. SCOEL used the summary epidemiology data to derive an OEL by extrapolating to 40 years, adjusting the value for a bias in the exposure assessment and dividing by an uncertainty factor of two. In both cases, the extrapolated critical risk estimates were based on exposures well-below the concentration range of the collected exposure data.

### Key findings from coffee roasting and packaging investigations

Virji et al. (14) investigated exposure-response relationships using data from cross-sectional exposure and health surveys from 17 coffee facilities. Personal exposures to diacetyl, 2,3-pentanedione, and their sum were assigned to participants using their work history information and a job exposure matrix developed using data from the exposure surveys (8). Exposure metrics calculated included the highest 95th percentile, cumulative, and average exposures. There was variation in the modeling results with different exposure metrics showing statistically significant associations with different measures of respiratory health. Some key findings for the exposure-response analyses were: (1) metrics calculated using a worker's whole work-history in coffee production showed associations with certain health outcomes; (2) increases in the highest 95th percentile exposure metric were associated with decrements in continuous measures of lung function as well as certain categorical health outcomes; (3) average and cumulative exposure metrics were associated only with categorical health outcomes; (4) both diacetyl and 2,3-pentanedione were associated with certain health outcomes, although not always the same ones; (5) the sum of diacetyl and 2,3-pentanedione captured all the significant associations that were observed in separate analyses of each α-diketone. The α-diketone-related respiratory abnormalities occurred in both flavoring and non-flavoring workers, however in the flavoring workers all exposure metric means were numerically higher, the model coefficients were larger, and the associations were more consistent across both α-diketones. Additionally, Harvey et al. (15) described a case of advanced lung disease in a coffee worker who had worked in the flavoring room and coffee grinding area of a coffee facility for a number of years. Although the biopsy findings were not typical of obliterative bronchiolitis, the authors noted that lung pathology may vary in flavoring-related lung disease. Results of full-shift personal air-samples collected in the flavoring and grinding areas on other workers indicated diacetyl levels of 41–421 ppb and 2,3-pentanedione levels of 22–276 ppb – well-above the NIOSH RELs (i.e., an 8 to 84-fold difference for diacetyl and a 2 to 30-fold difference for 2,3-pentanedione).

### Potential to close gaps and revisit assumptions?

Given the varied approaches that have been taken in assessing the human health risk of exposure to diacetyl, it is certainly of interest to consider whether the availability of new exposure and health data in real-world settings may reduce uncertainties and inform assumptions.

As noted above, a primary source of divergence between published final, proposed, candidate or recommended OELs for α-diketones stems from the decision to use animal or human data in conducting quantitative risk assessments. When available, the use of high-quality human data is preferable to animal toxicological data in conducting quantitative human health risk assessments. One of the main arguments raised against using observational epidemiologic data in conducting quantitative risk assessments is uncertainties determining causality based on concomitant exposures to other respiratory hazards or other confounding variables, both recognized and unrecognized. However, there is a compelling argument for the use of human data in the derivation of an OEL for diacetyl given the strong and consistent association between diacetyl exposure and adverse respiratory outcomes in epidemiological investigations. In addition, these associations are supported by animal toxicological studies demonstrating a clear dose-response relationship between diacetyl and respiratory morbidity.

Virji et al. (14) demonstrated a statistically significant association between exposure to both diacetyl and 2,3-pentanedione and decrements in lung function and abnormalities. Of interest, the most consistent associations between exposure to α-diketones (diacetyl, 2,3-pentanedione and the sum of the two) and lung function abnormalities were observed using the highest 95th percentile exposure during the on-average 4-year work-history, which is described by the authors as a surrogate of peak exposures. Nonetheless, evidence is also presented of significant positive associations between both average and cumulative exposures and lung function abnormalities. These results are generally consistent with previous epidemiological findings from exposures to added flavoring chemicals in which workers were exposed to much higher concentrations of diacetyl over longer periods of time. While Virji et al. (14) posit that the lack of associations between forced expiratory volume in one second (FEV_1_) or the ratio between FEV_1_ and forced vital capacity (FEV_1_/FVC) and average or cumulative exposure in their analysis precludes a direct comparison with the analysis presented in the NIOSH criteria document (1), it is worth noting that among flavoring workers, the authors reported negative associations between cumulative exposure to diacetyl, 2,3-pentanedione and their sum, and the percent predicted FEV_1_, albeit with a small sample size (*n* = 71).

Quantitative risk assessments for diacetyl have generally assumed that the adverse respiratory effects of exposure to diacetyl are driven by cumulative exposures over a working lifetime. Although NIOSH also recommended short-term exposure limits at levels 5 times the REL to protect against toxicity from 15-min peak exposures, the criteria document highlights the need to better characterize peak exposures, generally defined as brief or intermittent exposures to high concentrations, that may be relevant to human health risk (1). As previously noted, long-term cumulative exposure is generally the most used predictor of risk for chronic health outcomes; however, observed effects may in fact be more directly related to other dose metrics. For example, health effects may be driven in part by unmeasured peaks over time, particularly for chemicals that are metabolized and/or eliminated without overwhelming homeostatic responses at low levels of body burden. In an evaluation of histopathologic changes to the respiratory epithelium of rats following continuous and short-term pulsed exposures to diacetyl designed to result in similar time-weighted average exposures, Hubbs et al. (16) reported similar effects from both exposure regimens, suggesting that additional studies of short-term exposures is warranted. Epidemiologic investigations used to derive the NIOSH REL for diacetyl measured or estimated full shift TWA exposure concentrations, and did not have sufficient data to investigate the extent to which peak exposure concentrations correlate with the observed associations between exposures to α-diketones and adverse health outcomes. However, these investigations and subsequent analyses do provide some evidence that peak exposures may play a role in the associations observed between cumulative exposure to diacetyl and 2,3-pentanedione and respiratory morbidity. These findings are certainly of interest and merit further investigation, though are far from sufficient to substantially alter the current quantitative risk assumptions that health effects are driven by cumulative exposures.

## Discussion

To date, the epidemiologic evidence of associations between diacetyl and respiratory morbidity has largely come from occupational exposures to flavoring additives, while associations between 2,3-pentanedione and respiratory outcomes have not previously been evaluated in epidemiologic investigations. In the studies included as part of this research topic on exposures and respiratory health in coffee workers (7), exposures to diacetyl and 2,3-pentanedione from both flavoring and non-flavoring coffee workers have been characterized, offering the potential to inform our understanding of the impact of both diacetyl and 2,3-pentanedione on respiratory morbidity. These peer-reviewed articles represent a significant contribution to the body of knowledge regarding respiratory effects of exposure to relatively low levels of α-diketones emitted naturally from roasting and grinding coffee as well as from added flavoring chemicals in processing flavored coffee. Beckett et al. (2) posits that an OEL as low as 5 ppb is not practical in protecting health, citing studies that have measured higher concentrations of diacetyl as a result of brewing coffee. It would appear that this assertion is based solely on the observation that some individuals are frequently exposed to concentrations at or above the NIOSH REL, while discounting the possibility that over time these exposures may result in small decrements in lung function among some individuals. The health effects resulting from exposures to the relatively low levels of diacetyl observed in these investigations (average and highest 95% percentile personal TWA exposures below 25 and 100 ppb, respectively) have not been extensively or systematically studied. However, the analyses conducted by Virji et al. (14) have provided evidence that exposures to these levels of diacetyl and 2,3-pentanedione emitted (1) naturally from coffee roasting and grinding, and (2) from flavorings added in processing flavored coffee, may be associated on average with small decrements in lung function and further, in at least one case, with clinical disease as described by Harvey et al. (15).

In evaluating how this information might inform quantitative risk assessment of chronic low exposures, it is illustrative to examine the potential impact on the NIOSH risk assessment. In the NIOSH quantitative risk assessment for diacetyl, the risk to an individual worker exposed to the REL over the entirety of their working life is estimated to be 1 in 1,000 for developing an exposure-induced decrement in lung function as defined as an FEV_1_ below the 5th percentile (lower limit of normal). Workers exposed for less than working lifetime are predicted to be at lower risk. One difficulty in conducting occupational epidemiology is when exposure concentrations are near the REL (5 ppb in the case of diacetyl and 9.3 ppb in the case of 2,3-pentanedione), many workers would need to be studied for a very long time to quantitatively describe the risks, which is rarely feasible; therefore, studies with higher exposures are often used to estimate risks. However, when additional epidemiology studies describe dose-rate effects, such as decrements in lung function associated with peak exposures, this information can be used to expand our understanding of the mechanism of toxicity and can provide some insights into what types of exposures may be critical for workers' health. These considerations would similarly inform other quantitative risk assessments based on cumulative exposures to diacetyl.

There will undoubtedly be differences of opinion in the extent to which this information can or should be used to quantify and/or interpret the risks of exposure to diacetyl and 2,3-pentanedione. In our view, the relatively small sample size with concentrations that are limited to the lower range of exposures may prove inadequate to support a full quantitative risk assessment. Further, the presence of both diacetyl and 2,3-pentanedione exposures makes it difficult to combine this dataset with the human data upon which the NIOSH and SCOEL assessments are based, which included only diacetyl exposure. Nonetheless, the insights gained from this research should be of use to occupational health professionals in evaluating and managing risks from exposure to diacetyl and 2,3-pentanedione, alone or in combination, in coffee processing facilities.

## Data availability statement

The original contributions presented in the study are included in the article, and further inquiries can be directed to the corresponding author.

## Author contributions

DJ proposed the concept and wrote the first draft. CW summarized details of published risk assessments for diacetyl. DJ, CW, and JC-G contributed to defining the scope of the work and designing the article. JC-G summarized key findings from recent investigations of exposures and respiratory endpoints among workers in coffee roasting and packaging facilities. All authors contributed to manuscript revision and review and approved the final submitted version.

## Funding

This work was supported by the National Institute for Occupational Safety and Health (NIOSH).

## Conflict of interest

The authors declare that the research was conducted in the absence of any commercial or financial relationships that could be construed as a potential conflict of interest.

## Publisher's note

All claims expressed in this article are solely those of the authors and do not necessarily represent those of their affiliated organizations, or those of the publisher, the editors and the reviewers. Any product that may be evaluated in this article, or claim that may be made by its manufacturer, is not guaranteed or endorsed by the publisher.

## Author disclaimer

The findings and conclusions in this report are those of the author(s) and do not necessarily represent the official position of the National Institute for Occupational Safety and Health, Centers for Disease Control and Prevention.
